# Feasibility and preliminary efficacy of training health workers in detecting Priority Mental Health Conditions among adolescents in rural South India

**DOI:** 10.1186/s40814-022-01215-9

**Published:** 2022-12-31

**Authors:** Archana Siddaiah, Krishnamachari Srinivasan, Veena Satyanarayana, Maria L. Ekstrand

**Affiliations:** 1 Community Health Department, St John’s Medical College Hospital, Sarjapur road, John Nagar, Kormangala, Bengaluru, 560034 India; 2 Department of Psychiatry, St John’s Medical College, Head, Division of Mental Health and Neurosciences, St John’s Research Institute, Bengaluru, 560034 India; 3 Department of Clinical Psychology, National Institute of Mental Health and Neuro Science, Bengaluru, 560034 India; 4 Department of Medicine, Division of Prevention Science, University of California, San Francisco, USA

**Keywords:** mhGAP, Adolescent mental health, Community healthcare workers, India

## Abstract

**Introduction:**

Half of all mental disorders start during adolescence, before 14 years. In India, the current prevalence of mental disorders in 13–17 years age group was 7.3%. Many gaps persist in the mental healthcare delivery through the national mental health program, the low psychiatrist population ratio being one of them. Community health workers can play an essential role in providing mental healthcare in such resource-constrained settings. The World Health Organization mental health gap action program (WHO mhGAP) is a widely studied mental health tool that health workers can use to identify mental disorders. The study’s aim was to test the preliminary efficacy of training healthcare workers (HCWs) in identifying mental health conditions among adolescents using modified WHO mhGAP modules.

**Methods:**

The feasibility study was carried out in two Primary Health Centers (PHCs) in rural Bengaluru. Study had two components: (1) training of HCWs on adolescent mental health and (2) detection of selected priority mental health conditions among adolescents by trained HCWs. HCWs were trained in five adolescent mental health conditions using a training manual and modified WHO mhGAP modules that excluded emergency presentations and management sections. Pre- and post-training assessments were carried out. A sample of 272 adolescents attending PHCs were assessed for any mental health condition by HCWs using mhGAP modules. A sub-sample of adolescents and all adolescents identified by HCWs with a mental health condition was interviewed by the investigator to validate the diagnosis. Qualitative interviews were carried out with participating HCWs to understand the acceptability of the intervention, acceptability, and barriers to training in identifying mental health conditions among adolescents

**Results:**

A total of 23 HCWs underwent training. There was a significant increase in the mental health knowledge scores of HCWs post-training compared to baseline (*p* value <0.001). Out of 272 adolescents, 18 (6.8%) were detected to have any mental health condition by HCWs as per the modified WHO mhGAP modules. A sample of 72 adolescents consisting of all adolescents identified with a mental health condition by HCWs and a random sample of adolescents without any diagnosis were validated by the research investigator (AS). There was a good agreement between diagnosis by health workers and the research investigator with a Cohen’s Kappa of 0.88. Four themes emerged from the qualitative analysis.

**Conclusions:**

Training was effective in improving the knowledge of HCWs. There was a good agreement between trained HCWs and the investigator in detecting adolescent mental health conditions using modified mhGAP modules. The modified WHO mhGAP can thus be used by trained non-specialist HCWs to screen for adolescent mental health conditions in primary health centers.

## Key messages regarding feasibility

- What uncertainties existed regarding the feasibility?

The uncertainty that existed was the feasibility of training front-line workers in public health facilities to use the WHO mhGAP modules, which were not part of any government programs to detect adolescent mental health conditions.

- What are the key feasibility findings?

The key finding was that front-line workers could use the modified WHO mhGAP modules effectively to screen mental health conditions after brief and minimal training.

- What are the implications of the feasibility findings for the design of the main study?

The findings from this study could form the basis for designing and carrying out a randomized controlled intervention trial to improve adolescent mental health outcomes using non-specialist health personnel linked to primary health centers.

## Introduction

India has the largest adolescent population globally; with every fifth person being between 10–19 years of age [1, 2]. Adolescents experience profound physical, social, and psychological changes, making good mental health paramount for overall wellbeing. Mental health is a state of wellbeing in which an individual realizes his or her abilities, copes with everyday stresses of life, works productively, and contributes to his or her community [3]. Adolescents are exposed to complex familial, cultural, societal, economic, and environmental factors. These include poverty, violence, sexual abuse, migration, gender inequality, and humanitarian emergencies, which can affect their mental health [3, 4]. Mental health problems affect 10–20% of children and adolescents, accounting for a large portion of the global burden of disease [5–7]. Half of all the mental health disorders begin during the adolescent years [3, 8]. Worldwide, depression, and suicide are among the top causes of illness and disability among adolescents [9–12]. In India, as per the National Mental Health Survey (NMHS), the current prevalence of mental disorders in the age group, 13–17 years, was 7.3% [13]. Among 13–17 years old, anxiety disorders were the most common mental health condition (3.6%), followed by depressive disorders (0.8%) [14]. Poor mental health among adolescents can negatively affects one’s life management skills, interpersonal relationships, school performance, and productivity [15, 16]. The adolescent period offers an opportunity for interventions to prevent mental health disorders and promote good mental health to influence their lives positively. Despite this, the needed child and adolescent mental health services are not fully met anywhere in the world [7]. Lack of resources, stigma, and other barriers make it difficult for adolescents to access mental health care [17].

Worldwide, only 1% of the global health workforce provides mental health care with an expenditure of less than $ 2 per capita per year [4, 18]. In 2020, there were 0.4 psychiatrists per 100,000 population in lower middle-income countries (LMICs) compared to more than eight psychiatrists per 100,000 population in high-income countries [19]. India has 0.75 psychiatrists per 100,000 population [11]. Disparities exist in the mental health inpatient and outpatient services for children and adolescents, which are more pronounced in LMICs [20]. The WHO Comprehensive Mental Health Action Plan 2013–2030 recommends providing comprehensive mental health services in community-based settings [21]. In such a context, primary healthcare workers (HCWs) could play an essential role in providing mental health services in the community. India launched the district mental health program (DMHP) in 1996. DMHP offers clinical services, outreach services in the primary health centers (PHCs), community health centers, and sub-district hospitals. The program also provides mental health training to HCWs and disseminates information about mental health conditions to the lay public [22]. However, many gaps persist in the mental healthcare delivery through DMHP such as lack of trained manpower, underutilization of services [23].

In LMICs, training primary care providers to deliver child and adolescent mental health services lags behind services available for adults [17]. The WHO has developed an intervention tool for priority mental, neurological, and substance use disorders called Mental Health Gap Action Programme (mhGAP) to be used by trained non-specialist health providers in resource-constrained settings. The mhGAP focuses on providing evidence-based tools to strengthen the services for common mental health disorders. The revised mhGAP has guidelines for diagnosing and managing mental health conditions among children, adult, and geriatric populations [18]. With the uptake in over 90 countries, mhGAP is a widely studied mental health tool [24]. Most of such studies have been conducted in African and South-East Asian countries and mostly among adult populations, with only a few being conducted among pregnant adolescents, people with tuberculosis/HIV or those affected by humanitarian conflicts [25]. To the best of our knowledge, no studies have looked into the feasibility of implementing mhGAP modules for the adolescent population in rural India. The main objective of the present study was to assess the feasibility of training HCWs (such as staff nurse, pharmacist) in detecting mental health conditions among adolescents visiting the study PHCs in rural Bengaluru using mhGAP modules. We also wanted to test the diagnostic agreement between the trained health workers and the investigator.

## Methods

### Study design: feasibility interventional study

#### Study setting

The present study was conducted in two PHCs under the sub-district Anekal, namely Chandapura, and Dommasandra located in the state of Karnataka, India. The population density of Anekal was 561/km^2^ and had 13 PHCs and the adolescent population of Bengaluru district was 2 million [26, 27]. Each PHC was staffed by 10–15 HCWs. These two PHCs were selected based on their location within the rural field practice area of the study institution (St John’s Medical College). Since these centers comes under the public health system, they are similar to the other 11 PHCs in terms of their rural location, population covered, staffing pattern, and availability of infrastructure and resources.

#### Study population

Our study had two components: (1) training and capacity building of HCWs with respect to basic information on adolescent mental health conditions and (2) detection of selected mental health conditions among adolescents by trained HCWs. For the study, HCWs who had more than 12 years of schooling and who had worked in the study PHCs for more than 1 year were eligible to participate. HCWs included staff nurses, multi-purpose health workers, laboratory technicians, pharmacists, and medical officers working in the study PHCs. Adolescents of both gender, aged 10–19 years, residing in the Dommasandra and Chandapura villages, and availing of out-patient services in the study PHCs were eligible for the second component of the study.

#### Training manual and modified mhGAP modules

For training HCWs, an adolescent mental health training manual was prepared and translated into the local language (Kannada). The training manual was prepared using the WHO mhGAP and the Training Manual for Community Health Workers on Reducing risk factors for non-communicable diseases in primary care developed by the National Institute of Mental Health and Neurosciences, Bengaluru [28]. The WHO mhGAP has modules for 11 conditions. Each module has an assessment, management, and follow-up sections for each condition. In the assessment section, a psychiatric diagnosis is arrived at using an alogorithm based on the elicited symptoms. The other two sections deal with appropriate therapeutic interventions specific to each condition, including pharmacotherapy, psychotherapy, and follow-up. For training, we selected five modules based on the mental health conditions commonly encountered among adolescents. These were depression, self-harm/suicide, substance use, behavioral disorders, and anxiety disorders.

#### Modifications done for mhGAP modules

Each module was five pages long, including the plan for managing that particular condition. Since our outcome of interest was the detection of mental health conditions by trained HCWs, we excluded the management and follow-up sections from each module. We removed questions about emergency presentations such as alcohol intoxication or opioid withdrawal from the substance use disorders module since such presentations were rare in the PHC (as per the inputs by the PHC medical officer). Modifications were done by the research team after consultations with child and adolescent psychiatrists. We converted each module into a concise single-page algorithm to make it user-friendly for the HCWs. Since the mhGAP did not have an anxiety disorder module, it was prepared by the study team after receiving input from two psychiatrists. Face validation was carried out by an expert child psychiatrist. All modules were translated into Kannada. After pre-testing among Anganwadi workers (AWW) (primary level workers involved in child and adolescent health services), these modules were finalized. AWW were selected since they fit the cultural and demographic profile of our study participants. Before pre-testing, a mental health awareness session was conducted for AWW. Pretesting was done by briefing AWW about the modules by the investigator (AS). Following the briefing, AWW were asked to go through the modules for ease of understanding of question, wordings, and comprehension. They administered the selected modules to a group of adolescents visiting the anganwadi center to check whether the questions were understood by the adolescents and the time required for individual assessments.

##### Training and hands-on experience

HCWs were trained for 10 h over 3 days by the investigator (AS) and a child/adolescent psychiatrist in both of the PHCs. Each training session included didactic lectures and case discussions. A training guide, flip charts, audio-visual aides, and role plays were used during the training sessions. After completing training, HCWs were given hands-on experience in applying the modules in the PHC. Hands-on experience involved two components for each condition. These were introduction of the condition using case scenarios in which common presenting symptoms were discussed. This was followed by assessment of the mental health condition using role play (Table 1).

**Table 1:** Details of training topics, learning objectives, and the methods

Day	Topic	Learning objective	Method
1	Introduction to adolescent issues	To define “adolescent” age group To determine the importance of adolescent mental health issues To highlight common mental health problems faced by adolescents To build rapport with adolescents	Didactic session using PowerPoint presentation (PPT) Group discussion Role play
	Behavioral disorders	To define attention deficit hyperactivity disorder (ADHD) To diagnose ADHD based on signs and symptoms among adolescents	Didactic session using PPT Group discussion Case study
	Depression	To understand depression among adolescents To diagnose depression among adolescents To outline various means of managing depression	Didactic session using PPT Case study Clarification of myths and misconceptions
	Substance abuse	To list different types of substance abuse among adolescents To diagnose substance abuse among adolescents To outline various means of managing substance abuse	Didactic session using PPT Case study Clarification of myths and misconceptions
2	Self-harm and suicide	To understand the difference between self-harm and suicide To identify acts or thoughts of suicide/self-harm among adolescent To outline various means of managing and preventing self-harm and suicide	Didactic session using PPT Case study Clarification of myths and misconceptions
	Anxiety disorders	To define anxiety disorder To identify the signs and symptoms of anxiety disorders To outline various means of managing anxiety disorders	Didactic session using PPT Case study Group discussion
	Application of modules	To understand mhGAP intervention guide To promote mhGAP use by trained HWs To perform assessments for selected mental health conditions using mhGAP modules	Didactic session using PPT Demonstration
3	Field testing of modules	To demonstrate how to use modules to detect selected mental health conditions among adolescents	Consultating adolescents attending PHCs

#### Pre- and post-training assessments of the HCWs

In order to test the baseline knowledge of HCWs about adolescent mental health issues, a pre-training assessment was conducted. A pilot-tested, structured, and self-administered, 25-item multiple-choice questionnaire was used. Broad domains included knowledge of common mental disorders. Each correct response carried a score and questions which had more than one correct answer carried two scores. All questions were drawn from the topics covered in training. The questionnaire took 30 min to complete. A post-training assessment was also administered at the end of the last training day using the same questionnaire (Table 2).

**Table 2:** Sample questions used for pre and post-training assessments

Which is the correct statement about depression?	
a) Depression is a sign of weakness and not a medical problem	
b) Depression can happen to anyone	
c) Only mentally ill patients can get depression	
d) Once depressed, the patient will be depressed for life	
All of this is correct about anxiety disorders among adolescents except?	
a) Impairment in carrying out day to day activities.	
b) Can lead to poor social and interpersonal relationships	
c) This is not commonly seen among adolescents	
d) Stress reliever activities control anxiety disorders	

#### Community awareness and engagement

Information education and communication activities were conducted in the schools, colleges, and communities in the study PHC area to create awareness among adolescents about mental health issues and inform them about the services available as part of this project. An informative booklet prepared by the study team on adolescent mental health was distributed to all of the adolescents during these sessions. These activities were coordinated with the Panchayat members (i.e., the local administrative unit) and the respective school principals.

##### Assessment of adolescents by trained HCWs

Trained HCWs assessed all adolescents seeking care at their respective PHCs for the five conditions using the modified mhGAP modules. Each adolescent was assessed by only one HCW. HCWs received $10 as a monetary incentive for their time doing this activity. Adolescents received a gift kit containing a pen and an information booklet on adolescent mental health. All adolescents identified as having a mental health condition by the trained HCWs, and a random sample of adolescents without any psychiatric diagnosis were interviewed by the investigator (AS) to validate and check for a diagnostic agreement. Assistance of a psychiatrist was sought telephonically to arrive at a consensus if there were any disagreements in the diagnosis between the HCWs and the investigator (AS). The investigator (AS) and the two research assistants coordinated the data collection process in the PHCs. Data were collected from April 2019 to November 2019.

#### Qualitative methodology

Two focus group discussions were conducted in order to understand HCWs’ experiences in providing adolescent mental health care using the mhGAP modules and its acceptability. The groups consisted of a convenient sample of six to eight trained HCWs from both the PHCs. Two key informant interviews were conducted with the medical officer-in-charge and staff nurses of the PHCs. An interview guide was prepared with open-ended questions and translated into the local language (Kannada). Consent was obtained from the participants for audio-recording the discussions. A female community health physician (AS) with experience in qualitative research moderated the discussions. De-identification was done by allotting unique study numbers to participants. Discussions were conducted in a quiet room within the PHC and at a time convenient to the participants. Each session lasted for 20–40 min. At the end, the moderator summarized the findings and verified them with the participants for accuracy.

##### Sample size

**Fig. 1:**
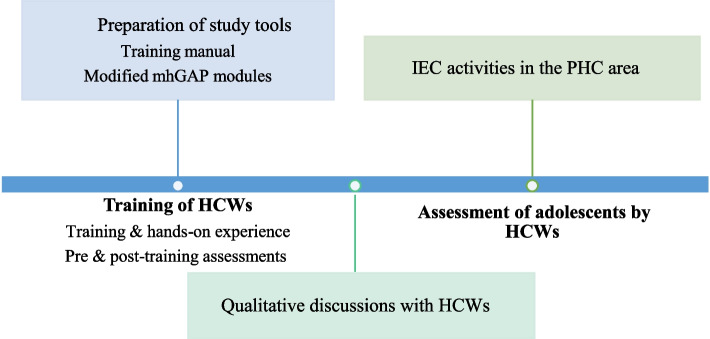
Schematic representation of study components

The sample size calculation was done based on the study outcome, which is an agreement analysis on the diagnosis of the mental health condition between the trained HCWs and the investigator. Assuming an agreement (kappa value) of 0.7 against null value of 0.50 with 90% power, the required sample size was 231 adolescents [29]. All 23 trained HCWs were invited to participate in the qualitative discussions (Fig. 1).

### Confidentiality and ethical issues

Ethical approval was obtained from the Institutional Ethics Committee at St John’s National Academy of Health Sciences (reference number 131/2018). Permission to conduct the study was obtained from the Medical Officer of the respective PHCs. Participants were identified in the study PHCs. A separate informed written consent was administered to all HCWs and adolescents. For adolescents below 18 years, a written assent was obtained from the adolescents along with written consent from their parent/guardian. Adolescents identified as having the selected mental health condition were referred to a nearby psychiatry clinic for further evaluation and management.

### Statistical analysis

Data were entered in Epi data, version 1.4, and analyzed in Stata 12. The difference in pre-training and post-training assessment scores of HCWs was tested using the Wilcoxon signed-rank test. Agreement between HCW and investigator on the presence or absence of a mental health condition was assessed by calculating Kappa with 95% confidence intervals. Audio recordings of the qualitative discussions were transcribed ad verbatim and translated into English. For analysis, transcripts were manually coded and categorized in Microsoft excel. Analyses were done using thematic framework approach [30]. Example codes included HCW’s experience in working with the public health system and inadequate or non-existence of adolescent mental health services. The analyses were done by the first author (AS) with the help of a research assistant. Transcripts were translated into English for ease of doing analysis. Audiorecordings were repeatedly listened to in order to ensure there was no loss of information during the process. Under each category, contents of the data were abstracted. Different themes were derived after summarizing these abstractions.

## Results

All eligible HCWs (n-23) consented to participate and received training. The pre-training assessment mean score was 23.2 (SD 5.1), and the median was 23 (IQR 18-29) out of 35. The post-training assessment mean score was 28.4 (SD 4.5), and the median score was 28 (IQR 24–33). There was an increase in the mental health knowledge scores of HCWs post-training and the pre- and post-difference in the median score was significant (*p* value <0.001). Out of 272 adolescents 18 (6.8%) adolescents were identified to have any mental condition by HCWs as per the modified WHO mhGAP modules. The investigator (AS) verified the diagnosis of a random sample of 72 adolescents without any diagnosis and all 18 adolescents identified with mental conditions (Table 3). There was a good agreement between diagnosis by HCWs and the investigator (AS) with a Cohen’s Kappa of 0.88 (Table 4).

**Table 3:** Classification of adolescents by trained HCWs and investigator (*n*=72)

Sl no	Conditions classified	HCWs	Investigator
1	No diagnosis	54	56
2	Behavioral disorders	1	0
3	Depression	4	4
4	Suicide/self-harm	4	3
5	Substance abuse	7	6
6	Anxiety disorders	2	2
	Total	72	72

**Table 4:** Agreement analysis analysis between trained HCWs and investigator (*n*=72)

HCWs	Investigator		Total
	Mental health condition present	Mental health condition absent	
Mental health Conditions present	16	2	18
Mental health Condition absent	0	54	54
Total	15	56	72
**Cohens Kappa 0.88 (*p*value <0.001) CI (0.63–0.98)**			

### Qualitative results

Two focus groups and three in-depth interviews were conducted involving 18 trained HCWs. Four themes emerged from the qualitative analysis.

1. Even though challenging, serving in public health facilities left HCWs satisfied because it helped the underserved populations: HCWs stated that working in the public health system left them frustrated due to huge workload, lack of respect from patients, and less job satisfaction. However, some HCWs also described how working for a long time close to the communities had made them confident in assessing the health needs of the people and able to deliver appropriate care. For some HCWs, helping under-served populations was rewarding.
  “Many patients who come here are poor and they cannot afford services at private healthcare facility.”- Female participant -08
  “We have three to ten years of working in this center. Since we are working in the field and going to community and interacting with them, we understand what kind of services they require.”- Female participant -01
2. Adolescent mental health issues were perceived to be essential; however, HCWs found adolescents not open about their issues: Participants highlighted the importance of mental health problems among adolescents. However, many adolescents were not ready to share their emotional problems with HCWs. This was one of the challenges experienced by the participants. This was true for clinic or community settings.
  “Adolescent health is very important as they are exposed more to society than us.”
  “Adolescents even when they experience some symptoms they will not share it with others openly.” -Male participant -05
3. Improved confidence of HCWs in dealing with mental health issues following training: With an effective training and dedicated time, HCWs expressed confidence in using modules to detect adolescent mental health conditions. HCWs used the modules and referred to the training manual whenever they required more information.
  “After training, we came to know how to deal with adolescents. How to ask for their issues and how we can help them and advice about their issues.” -Female participant -04
  “It (training) was very informative and the manual which was given provided lot of information. Especially for our field staff.”- Female participant-01.
  “If we come across such patients now, we can give them counseling and refer to a doctor.”- Male participant-10
4. Lack of time was a significant obstacle: Often, HCWs were disturbed by other patients or medical emergencies when they were doing assessments of adolescents in the PHC. HCWs stated that they were willing to provide mental health services. However, routine work schedules including fieldwork, patient care, and reporting made it difficult for them to provide mental health services. In addition, there was a lack of dedicated counseling services, and an inconsistent supply of psychotropic medications.
  “When we were doing assessments in the PHC, patients used to come and we had to stop carrying out assessments in between to give treatment to emergency patients.”- Female participant -04

## Discussion

Our study results suggest that after a brief training, PHC HCWs were able to identify common adolescent mental health conditions. This finding has important policy and clinical implications for a country like India, which has very few trained mental health professionals [14], showing that trained HCWs can bridge the existing gap in mental health diagnoses and services. One of the objectives of the WHO Comprehensive mental health action plan 2013-30 is to provide comprehensive, integrated, and responsive mental health services in community-based settings [21]. This is relevant for adolescent patients as they benefit the most when health services are adolescent-friendly and easily accessible. This is where the implementation of mhGAP program in primary care by non-specialists is highly relevant. The WHO mhGAP has been used by program managers, NGOs, clinicians, researchers, and academicians as part of training programs, clinical implementations, country contextualizations, and forecasting economic models [31, 32]. The effectiveness of the mhGAP intervention on various mental health conditions under different circumstances has also been tested using randomized controlled trials (RCT) [33–36]. Studies examining characteristics of this training have reported that mhGAP can be successfully implemented in undergraduate and post-graduate medical courses as well as in nursing curricula. The involvement of psychiatrists in such programs led to an improved acceptance and successful implementation of mhGAP program in academic institutions [37, 38]. In comparison to such studies, our study assessed the partial implementation of mhGAP by HCWs. Future research should examine the management of adolescents with mental health conditions using mhGAP modules.

Findings from studies assessing the clinical implementation of mhGAP have also been promising [34, 39–42]. A pilot test of mhGAP modules among Kenyan primary health workers reported improved knowledge scores following training. One of their study topics was child and adolescent mental health problems; however, the authors did not assess the clinical usefulness after training [43]. In a pilot study conducted in Kenya, the authors successfully trained nurses and clinical officers and supervised the validity of diagnosis and outcomes, such as decrease in disability, seizure control using the mhGAP guide. In that study, depression was the most common condition diagnosed by the trained nurses [40].

In our study, we included a mix of PHC staff and field staff, including staff nurses, pharmacists, lab technicians, data entry operator, field workers, supervisors, and medical officers. This is similar to the populations selected in studies that have used mhGAP modules to train HCWs. A cross-sectional multi-site study involving 15,078 adults, the reported positive predictive value for traditional healers to screen common mental conditions using mhGAP was 72–95%. The study authors also reported good agreement between screening using the mhGAP by different service providers and independent diagnostic validation using Mini International Neuropsychiatric Interview for most of the conditions [41]. Similarly, in our study, we found good agreement between HCWs and the investigator in identifying selected mental health conditions among adolescents; however, due to the small sample size, we did not have sufficient power to evaluate the agreement between different categories of service providers. Going forward, it will be useful to evaluate such differences among HCWs in terms of their competence in detecting mental health conditions. Those showing greater competence could then be prioritized for delivering mental health services. In the qualitative discussions, HCWs reported difficulty eliciting sensitive information from adolescents. This also emerged as one of the themes during the analysis. Even though building rapport with adolescents was one of the training topics, we think this module needs further strengthening, to help HCWs become more effective in engaging with adolescent clients, despite multiple demands on their time.

One of the challenges in the implementation of such interventions within the health system is the involvement of strategic stakeholders. Some studies had shown that implementing the mhGAP modules was feasible when multiple stakeholders including community members were involved [43, 44]. Our engagement was limited to involving the local administrative units for IEC activities. But we could not involve stakeholders responsible for delivering mental health servies in the PHCs. A greater involvement of policy makers and health administrators would have given us more insights into the programmatic challenges of delivering mental health health services to adolescents at the PHCs. The qualitive analysis revealed some of the important challenges for HCWs working at the PHCs, particularly lack of time. Policymakers need to be sensitized about the issues related to working dynamics of primary-level HCWs. This will help to formulate policies that can facilitate HCWs to deliver adolescent-friendly mental health services.

### Strengths and limitations

The involvement of psychiatrist in the training programs as well as inclusion of a diverse group of HCWs and local administrative units were some of the strengths of our study. Some of the HCWs included in the study had undergone mental health training from DMHP in the past, which may have affected their knowledge scores. However, none of them had received training on identifying or diagnosing adolescent mental health problems. Due to time constraints, we were unable to contact other stakeholders such as policymakers and community members to seek their opinions on issues linked to adolescent mental health problems. This would have given more insight into the mental health needs and possible ways to deliver mental health services to adolescents.

### Implications

The findings need to be replicated in future studies with a larger sample size. Testing mhGAP based interventions for rural adolescents using RCT can be considered. The study findings are applicable to other PHCs across the state. Economic evaluations in terms of the cost-effectiveness of adolescent mental health interventions are needed to guide mental health policies. Research looking at integrating other existing adolescent-specific programs such as adolescent-friendly health clinics (AFHC) (for reproductive health and counselling for mental conditions) can also be explored. Such collaboration between AFHC and DMHP can support HCWs in early identification and referral to specialists thereby improving adolescents’ uptake of mental health services.

## Conclusions

We found that brief mental health training improved knowledge of adolescent mental disorders among HCWs working in PHCs and can build the capacity of health workers to deliver targeted mental healthcare. A significant agreement was observed between trained HCWs and the investigator in detecting adolescent mental health conditions using modified WHO mhGAP modules. This showed that the WHO mhGAP can be an effective tool to screen for adolescent mental health conditions by trained non-specialist HCWs. In the interest of time, it is best to target one or two most prevalent mental health conditions in the community and train primary-level HCWs to address those conditions. By this, the workload on HCWs will not be excessive. HCWs must also be trained in “therapeutic” skills such as communication skill, empathy, and non-judgemental attitudes, as these are critical while dealing with adolescents. Such supportive capacity-building programs will help fill a critical gap in delivering mental health services to adolescents.

## Data Availability

Data will be made available for interested researchers based on suitable request with the corresponding author.
